# The DoMYB102–*DoUGT71K2* Regulatory Module Mediates Heat-Induced Flavonoid Glycosylation in *Dendrobium officinale*

**DOI:** 10.3390/biology15151330

**Published:** 2026-08-06

**Authors:** Jingting Li, Yuxia Yang, Huaizhi Zhang, Xinwei Xie, Ruishu Niu, Xiaoyang Li

**Affiliations:** 1 School of Biological and Pharmaceutical Engineering, Lanzhou Jiaotong University, Lanzhou 730070, China; yuxia.yang@lzjtu.edu.cn (Y.Y.); xinwei.xie@lzjtu.edu.cn (X.X.); ruishu.niu@lzjtu.edu.cn (R.N.); xiaoyang.li@lzjtu.edu.cn (X.L.); 2 Institute of Genetics and Developmental Biology, Chinese Academy of Sciences, Beijing 100101, China; hzzhang@genetics.ac.cn

**Keywords:** *Dendrobium officinale*, glycosyltransferases, flavonoids, *DoUGT71K2*, gene expression profiling, high-temperature stress

## Abstract

*Dendrobium officinale*, a perennial medicinal herb rich in flavonoids, exhibits multiple pharmacological properties including antioxidant and hypoglycemic effects. However, its medicinal quality is compromised by various abiotic stresses, such as heat, cold, and osmotic stresses. UDP-glycosyltransferases play critical roles in both flavonoid glycosylation and plant stress tolerance. Here, we report the identification of a heat-responsive glycosyltransferase gene, *DoUGT71K2,* from *D. officinale* transcriptomes, and a characterization of its stress-induced expression and transcriptional regulatory mechanisms. Quantitative real-time PCR (qRT-PCR) showed that *DoUGT71K2* exhibits tissue-specific high expression in flowers, and its transcript abundance is strongly elevated in response to heat, cold, and abscisic acid (ABA) treatment. The direct promoter-binding and trans-activating function of DoMYB102 on *DoUGT71K2* was confirmed via dual-luciferase reporter and yeast one-hybrid (Y1H) assays. These results establish a *DoMYB102*–*DoUGT71K2* regulatory module that couples heat stress response with flavonoid biosynthesis, which provides a molecular basis for improving stress tolerance and medicinal quality in *D. officinale* through molecular breeding.

## Simple Summary

The accumulation of bioactive flavonoids in the medicinal herb *Dendrobium officinale* is highly susceptible to environmental stresses, particularly high temperature. Glycosyltransferases play critical roles in both flavonoid glycosylation and plant stress tolerance. In this study, we identified a heat-responsive glycosyltransferase gene, *DoUGT71K2*, from *D. officinale* and characterized its stress-induced expression profiles. *DoUGT71K2* was most abundantly expressed in flowers and was significantly induced by high temperature, low temperature, and abscisic acid (ABA). The transcription factor DoMYB102 directly binds to the promoter of *DoUGT71K2* and activates its transcription. This establishes a *DoMYB102*-*DoUGT71K2* regulatory module that links heat stress response with flavonoid biosynthesis. These results provide a scientific foundation for breeding *D. officinale* varieties with improved stress tolerance and enhanced medicinal quality.

## 1. Introduction

*Dendrobium officinale* Kimura et Migo is a perennial medicinal herb of the family Orchidaceae. It grows epiphytically on humid rocks or in cliff crevices and is widely distributed in Yunnan, Guangxi, Fujian, Zhejiang, and other provinces in southern China [1]. It is rich in polysaccharides, flavonoids, alkaloids, phenolic acids, phenanthrenes, and other bioactive components, exhibiting antioxidant, anti-inflammatory, antitumor, hypoglycemic, and gastrointestinal protective activities [1,2]. Among these components, flavonoids mainly include five subclasses: flavones, dihydroflavones, isoflavones, anthocyanins, and chalcones, most of which exist as glycosides [1,3]. The accumulation of these flavonoids is influenced by environmental conditions, such as temperature and light [4,5,6]. Marked diurnal temperature fluctuations and strong light intensity can promote flavonoid and other secondary metabolite accumulation. However, recreating these conditions in controlled cultivation is technically challenging, which restricts large-scale production and compromises medicinal quality uniformity [6,7]. Therefore, systematic elucidation of the transcriptional and regulatory profiles of flavonoid biosynthesis-related genes under abiotic stress is essential for enhancing the medicinal value of *D. officinale* and developing stress-tolerant varieties.

Glycosyltransferases catalyze the transfer of glycosyl moieties from uridine diphosphate sugars (e.g., UDP-glucose) to flavonoid backbones and thus generate flavonoid glycosides with improved structural stability and aqueous solubility [8,9]. UDP-glycosyltransferases (UGTs) are a major class of glycosyltransferases that function in the late biosynthetic steps of flavonoids and mediate glycosylation as a crucial post-modification process [10]. Most UGTs contain a conserved plant secondary product glycosyltransferase (PSPG) domain responsible for UDP-sugars binding [11]. Flavonoid biosynthesis originates from phenylalanine and proceeds to the core flavonoid skeleton through the sequential catalysis of phenylalanine ammonia-lyase (PAL), caffeoylshikimate esterase (CSE), 4-coumarate: CoA ligase (4CL), chalcone synthase (CHS), and chalcone isomerase (CHI). Subsequent glycosylation by UGTs generates a diverse array of flavonoid glycosides [11,12].

Glycosyltransferases are not only involved in the glycosylation of secondary metabolites but also contribute to plant stress tolerance [13]. Under abiotic stress conditions, glycosyltransferases regulate the glycosylation status of phytohormones, antioxidants, and osmotic regulators. This regulation alleviates oxidative cell damage and helps maintain osmotic homeostasis under drought, low temperature, high salinity, and other environmental stresses. Previous studies have revealed that UGTs play diverse roles in both primary and stress-responsive metabolism. Examples of UGT functional pleiotropy include AtUGT72B1-catalyzed monolignol glycosylation, which is essential for cell wall lignification in *Arabidopsis thaliana* [14], *OsUGT85E1*-mediated ABA elevation and reactive oxygen species (ROS) scavenging for drought stress tolerance in rice (*Oryza sativa* L.) [15], *MdUGT73AR4*-accelerated ABA biosynthesis and glycosylation for drought tolerance in apple (*Malus domestica*) [16], and *AtUGT86A1*-enhanced soluble sugar and ROS detoxification capacity for salt and drought tolerance in *Arabidopsis* [17]. Consequently, glycosyltransferases function as indispensable components of the plant stress defense system, which provides plants with enhanced adaptive potential to variable external environments.

In recent years, accumulating studies have emphasized the dual functions of glycosyltransferases in modulating flavonoid glycosylation and accumulation, as well as enhancing environmental adaptability in maize (*Zea mays*), tea (*Camellia sinensis*), and licorice (*Glycyrrhiza glabra*) [18,19,20]. However, the functional characterization of UGT in *D*. *officinale* under abiotic stress remains limited. Comprehensive investigation of UGT functions in this medicinal orchid under stress conditions is essential for unraveling the regulatory network of flavonoid biosynthesis. This will ultimately facilitate the improvement of stress tolerance in *D. officinale*.

In the present study, transcriptome screening identified a heat stress-induced glycosyltransferase gene, *DoUGT71K2*, and a transcription factor gene, *DoMYB102*, both of which were markedly upregulated upon high-temperature treatment. Subsequent homologous cloning and bioinformatic analyses indicated that *DoUGT71K2* functions in flavonoid glycosylation. Expression profiling further revealed that *DoUGT71K2* was highly expressed in flowers and responsive to various abiotic stresses, particularly heat stress and ABA treatment. Follow-up biochemical assays confirmed that DoMYB102 positively activates the transcription of *DoUGT71K2* by direct binding to its promoter. Together, these results reveal a MYB-UGT regulatory module that connects heat tolerance with flavonoid biosynthesis in *D. officinale,* which provides a candidate gene resource and a theoretical basis for breeding stress-tolerant cultivars.

## 2. Materials and Methods

### 2.1. Plant Materials and Sample Treatment

The *D. officinale* cultivar Honggan Ruanjiao used in this study was supplied by Jiujiurun Biotechnology Co., Ltd. (Ningbo, China). Two-year-old seedlings were grown in a greenhouse set at 26 °C under a 12-h photoperiod [21], using plastic pots filled with pine bark as the substrate and irrigated every three days until the substrate was fully saturated. For heat treatment, seedlings were incubated at 40 °C for 12 h. Young leaves at the 3rd to 6th positions distal to the shoot apex were collected and subjected to transcriptome sequencing at Shanghai OE Biotech. Co., Ltd. (Shanghai, China).

Fresh tissues of two-year-old *D. officinale* seedlings were collected to profile the expression of *DoUGT71K2* under various stress treatments: (1) for heat stress, young leaves, flowers, roots, and stems were collected after treatment at 40 °C, with untreated plants kept at 26 °C as the control [22]; (2) for cold stress, fresh leaves were harvested after treatment at 4 °C, with untreated plants kept at 26 °C as the control [23]; (3) for abscisic acid (ABA) treatment, fresh leaves were collected after foliar spraying with 100 μM ABA solution (300 mL per plant until run-off), while control plants were sprayed with the same volume of aqueous solution containing an equivalent concentration of ethanol (without ABA) [24]; (4) for osmotic stress, fresh leaves were collected after foliar spraying with 20% PEG 6000 solution (300 mL per plant until run-off), while control seedlings were sprayed with an equal volume of distilled water [24]. Time-course sampling was performed with three independent biological replicates per treatment at 0, 1, 3, 6, 9, 12, and 24 h. All harvested samples were immediately frozen in liquid nitrogen and stored at −80 °C.

### 2.2. RNA Extraction and cDNA Synthesis from D. officinale

Total RNA was extracted from fresh *D. officinale* leaves using a modified CTAB-LiCl method optimized for polysaccharide and polyphenol-rich plant tissues [25]. Briefly, leaf samples were ground in liquid nitrogen with steel beads and mixed with 700 μL of preheated (65 °C) CTAB extraction buffer supplemented with 60 μL β-mercaptoethanol. The mixture was vigorously vortexed, incubated at 65 °C for 10 min with intermittent gentle inversion (4–5 times), and extracted with an equal volume of phenol:chloroform:isoamyl alcohol (25:24:1, *v*/*v*/*v*). After a 10-min ice incubation and centrifugation at 12,000× *g* for 10 min, the supernatant was subjected to a second phenol–chloroform extraction. The resulting upper phase was combined with 1/2 volume of absolute ethanol and 1/3 volume of 10 mM LiCl, and precipitated overnight at −20 °C. The precipitate was collected by centrifugation (12,000× *g*, 4 °C, 10 min), washed twice with 70% DEPC-treated ethanol (with a 5 min centrifugation at 10,000× *g*, 4 °C between washes), air-dried on ice, and dissolved in 30 μL of 0.1% DEPC-treated water. RNA quality was assessed by 1% agarose gel electrophoresis, and concentration and purity (A_260_/A_280_) were measured using a NanoDrop spectrophotometer (Thermo Fisher Scientific, Wilmington, NC, USA). Total RNA was used to generate first-strand cDNA with the HiScript IV 1st Strand cDNA Synthesis Kit (Vazyme Biotech Co., Ltd., Nanjing, China), and the product was stored at −20 °C.

### 2.3. Gene Expression Profiling of DoUGT71K2

cDNA (50 ng·μL^−1^) synthesized from RNA extracted from leaf tissues subjected to heat, drought, and cold stresses, as well as from root, stem, flower, and leaf tissues, was used as the template for qRT-PCR. *Actin* was used as the internal reference gene. Primer pairs for *DoUGT71K2* were designed using NCBI Primer-BLAST, which uses Primer3 v2.5.0 (https://www.ncbi.nlm.nih.gov/tools/primer-blast/index.cgi accessed on 23 March 2025) and the Oligonucleotide Properties Calculator v3.36 (https://www.biosyn.com/Gizmo/Tools/Oligo/Oligonucleotide%20Properties%20Calculator.htm (accessed on 23 March 2025)). qRT-PCR was performed on a CFX Connect™ real-time PCR detection system (Bio-Rad, Hercules, CA, USA) using a qRT-PCR kit (Vazyme Biotech). The cycling protocol consisted of initial denaturation at 95 °C for 30 s, followed by 40 cycles of 95 °C for 10 s, 60 °C for 30 s. Melting curve analysis was performed under the conditions of 95 °C for 15 s, 60 °C for 60 s, and 95 °C for 15 s to validate product specificity. Relative transcript abundances were determined using the 2^−ΔΔCt^ method [26], with three biological replicates per treatment. All graphical representations were generated using GraphPad Prism 9.5.1 (https://www.graphpad.com/ (accessed on 1 January 2026)).

### 2.4. Amplification of Target Gene Fragments from D. officinale

The coding sequences (CDSs) of *DoUGT71K2* and *DoMYB102*, and the *DoUGT71K2* promoter region (approximately 2 kb upstream of the transcription start site) were amplified from *D. officinale* for subsequent functional assays. Gene-specific primers were designed using NCBI Primer-BLAST (which uses Primer3 v2.5.0) and the Oligonucleotide Properties Calculator (v3.36), based on the *UGT71K2* and *MYB102* homologous sequences of *Dendrobium catenatum* (Gene ID: LOC110110985 and LOC110098979) obtained from the NCBI database (https://www.ncbi.nlm.nih.gov/ (accessed on 1 January 2026)). The CDS fragments were amplified from cDNA, while the promoter fragments were obtained from genomic DNA, which was extracted from young leaves using a standard CTAB method. All PCR amplifications were performed using 2× Phanta Max Master Mix (Vazyme Biotech). PCR products were separated on 1.0% (*w*/*v*) agarose gels, excised, and purified using the Gel Extraction Kit (TIANGEN Biotech (Beijing) Co., Ltd., Beijing, China). The purified fragments were ligated into the pGM-T vector (TIANGEN Biotech (Beijing)) and transformed into competent *Escherichia coli* DH5α cells (TIANGEN Biotech (Beijing)) via heat-shock transformation. Positive clones were identified by PCR and verified by Sanger sequencing. Primer synthesis and Sanger sequencing services were provided by Tsingke Biotechnology Co., Ltd. (Xi’an, China). All primer sequences are listed in Supplementary Table S1.

### 2.5. Bioinformatics Analysis of DoUGT71K2

The nucleotide and amino acid sequences of *DoUGT71K2* were used for BLAST-based homology searches against the NCBI database. Physicochemical properties of the protein encoded by *DoUGT71K2*, including average hydrophobicity, molecular mass, and isoelectric point (pI), were computed using the ProtParam tool on ExPASy (https://web.expasy.org/protparam/ (accessed on 10 March 2025)). Transmembrane topology was predicted with TMHMM v2.0 (https://services.healthtech.dtu.dk/service.php?TMHMM-2.0 (accessed on 10 March 2025)). Secondary and tertiary structures were modeled using SOPMA (https://npsa-prabi.ibcp.fr/cgi-bin/npsa_automat.pl?page=npsa_sopma.html (accessed on 10 March 2025)) and SWISS-MODEL (http://swissmodel.expasy.org (accessed on 10 March 2025)), respectively. For phylogenetic analysis, homologous protein sequences were retrieved via BLASTp (https://blast.ncbi.nlm.nih.gov/Blast.cgi (accessed on 1 January 2026)), and used for phylogenetic tree construction with MEGA 11.0 (https://www.megasoftware.net/ (accessed on 1 January 2026)). Cis-acting regulatory elements within the 2.0 kb upstream region of the transcription start site were identified using the PlantCARE database (http://bioinformatics.psb.ugent.be/webtools/plantcare/html/ (accessed on 10 March 2025)). The binding sites of DoMYB102 were predicted using the JASPAR database (https://jaspar.elixir.no/ (accessed on 20 September 2025)).

### 2.6. Heat Map Analysis

Transcriptome sequencing was performed on RNA extracted from young leaves of *D. officinale* subjected to 40 °C heat stress for 12 h. Library construction and sequencing were completed by Shanghai OE Biotech. Co., Ltd. (Shanghai, China) on the DNBSEQ-T7 platform. Raw reads were filtered and mapped to the *D*. *catenatum* reference genome. Differentially expressed genes (DEGs) were identified with thresholds of |log_2_FC| ≥ 1 and *p* < 0.05. UGT and MYB transcription factor genes were extracted from the DEGs, and heat maps were generated using the online platform (https://www.bioinformatics.com.cn (accessed on 10 January 2025)) to visualize their expression patterns under heat treatment.

### 2.7. Dual-Luciferase Reporter Assay in Nicotiana benthamiana Leaves

To determine whether *DoMYB102* activates the *DoUGT71K2* promoter, a dual-luciferase reporter assay was carried out in *Nicotiana benthamiana* leaves. The *DoUGT71K2* promoter fragment was inserted into the reporter vector pGreenII 0800-LUC, and the CDS of *DoMYB102* was cloned into the effector vector pGreenII 62-SK, yielding pGreenII 0800-ProDoUGT71K2-LUC and pGreenII 62-SK-MYB102, respectively. All constructs were verified by Sanger sequencing. The recombinant plasmids were separately transformed into *Agrobacterium tumefaciens* GV3101 cells harboring the pSoup helper vector (Coolaber, Beijing, China) (OD_600_ = 1.0) and mixed at a 1:1 ratio (*v*/*v*). The mixed strains were co-infiltrated into the abaxial side of fully expanded leaves of 4- to 5-week-old *N. benthamiana* using needleless syringes. After infiltration, the plants were maintained in darkness for 6 h, then cultivated at 23 °C under a 16 h light/8 h dark photoperiod for 42 h. At 48 h post-infiltration, LUC and REN activities were measured using the Dual-Luciferase^®^ Reporter Assay System (Promega, Madison, WI, USA) on a multimode plate reader (PerkinElmer, Waltham, MA, USA). Relative promoter activity was determined as the LUC/REN ratio. Three biological replicates were performed for each experiment.

### 2.8. Yeast One-Hybrid (Y1H) Assay

The *BstBI*-linearized bait plasmid was transformed into yeast strain Y1HGold using the lithium acetate (LiAc)/polyethylene glycol (PEG) method, and integration of the bait construct into the yeast genome was verified by colony PCR. Transformed yeast cells were plated on SD/-Ura medium with increasing concentrations of aureobasidin A (AbA) to determine the AbA minimal inhibitory concentration (MIC) and to confirm the absence of bait self-activation. The resulting Y1HGold strain carrying the integrated bait construct (Y1HGold/DoUGT71K2-pAbAi) was then made competent for transformation with the prey construct. The p53/AbAi interaction provided in the Matchmaker^TM^ Gold Y1H system (San Jose, CA, USA) was used as the positive control, for which pGADT7-Rec53 was co-transformed into Y1HGold/p53-AbAi competent cells. The empty pGADT7 vector was used as the negative control, and pGADT7-MYB102 served as the experimental construct. Transformed yeast cells were plated onto SD/-Leu medium either without AbA or supplemented with the predetermined inhibitory concentration of AbA. Plates without AbA were used to assess transformation efficiency, whereas plates containing AbA were used to detect protein–DNA interactions. All solid plates were incubated at 30 °C for 2–3 days. Single colonies were picked from the SD/-Leu plate without AbA, resuspended in 100 μL of 0.9% NaCl solution, and serially diluted to 10^−1^, 10^−2^, and 10^−3^. A 5 μL aliquot of each dilution was spotted onto AbA-supplemented SD/-Leu medium, and the plates were incubated at 30 °C for an additional 2–3 days.

### 2.9. Statistical Analysis

Data were presented as mean ± standard deviation (SD) from three independent biological replicates. Statistical significance was assessed by one-way analysis of variance (ANOVA) followed by Tukey’s multiple comparison test. All statistical analyses were performed using SPSS 20.0 (IBM Corp., Armonk, NY, USA). Statistical significance was determined at *p* < 0.05.

## 3. Results

### 3.1. Screening of the UGT Gene and Amplification of the DoUGT71K2 CDS

Young leaves of *D. officinale* subjected to 40 °C heat stress for 12 h were harvested for transcriptome sequencing. Transcriptomic screening identified 25 differentially expressed UGT genes. Among these, 18 genes were downregulated, whereas seven were upregulated upon heat treatment (Supplementary Figure S1A). Of the seven upregulated UGT genes, *DoUGT71K2* exhibited the most pronounced upregulation under heat stress. Further GO annotation analysis revealed that this gene possesses UGT catalytic activity, suggesting a critical role in flavonoid glycosylation. Accordingly, *DoUGT71K2* was selected for further analysis of its induced expression pattern and transcriptional regulation.

**Figure 1:**
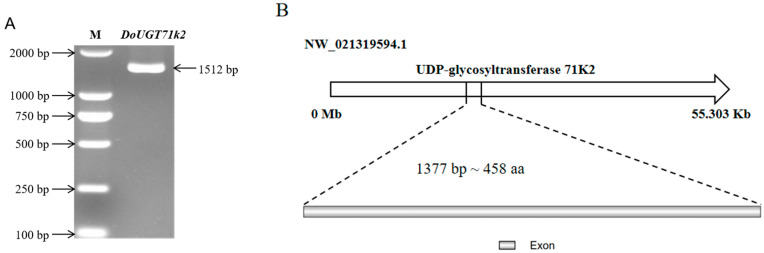
Cloning and structural characterization of *DoUGT71K2* from *D. officinale*. (**A**) Amplification of the *DoUGT71K2* CDS detected by 1.0% agarose gel electrophoresis. (**B**) Gene structure and chromosomal location of *DoUGT71K2.* M: DL2000 DNA ladder.

Gene-specific primers for amplifying the *DoUGT71K2* CDS were designed based on the homologous *UGT71K2* sequence of *D*. *catenatum* (Gene ID: LOC110110985). PCR amplicons were resolved on a 1.0% agarose gel, yielding a distinct 1512 bp target fragment (Figure 1A). Sequencing of the amplicon revealed that the full-length CDS of *DoUGT71K2* is 1377 bp, encoding a 458-amino acid protein.

Sequence alignment of the cloned CDS with the reference gene revealed variations at nine nucleotide positions, including two synonymous substitutions, one non-synonymous substitution, and six single-base deletions. The gene was mapped to scaffold NW_021319594.1 and was found to be intronless, containing only a single exon (Figure 1B).

### 3.2. Bioinformatic Analysis of DoUGT71K2

#### 3.2.1. Physicochemical Characteristics, Conserved Domain, Transmembrane Domain, and Secondary/Tertiary Structure of the DoUGT71K2 Protein

Physicochemical characterization of the DoUGT71K2 protein showed a molecular formula of C_2283_H_3575_N_613_O_643_S_19_, a predicted molecular mass of 50.5 kDa, and a theoretical pI of 6.26. Its instability index (49.29) suggests that the protein is unstable, while the grand average of hydrophilicity (GRAVY) value of 0.022 indicates that this protein is overall amphipathic with a marginal hydrophobic tendency.

**Figure 2:**
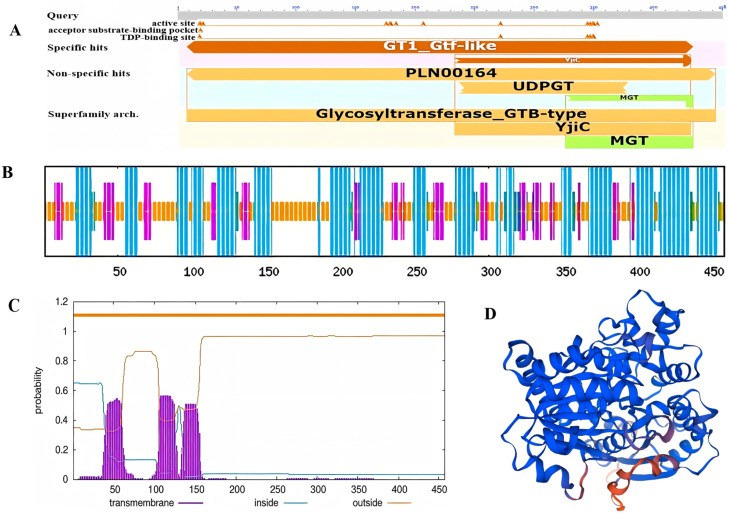
Domain and structure prediction of DoUGT71K2. (**A**) Conserved domain of DoUGT71K2. (**B**) Secondary structure prediction of DoUGT71K2. Blue: α-helix; purple: extended strand; orange: random coil; green: *β*-turn. (**C**) Transmembrane domain prediction of DoUGT71K2. (**D**) Tertiary structure prediction of DoUGT71K2.

Conserved domain prediction identified a conserved GT1-Gtf-like UDP-glycosyltransferase domain, which confirms that DoUGT71K2 belongs to the GT1 glycosyltransferase family (Figure 2A). Transmembrane domain prediction revealed that this protein lacks a typical transmembrane domain, indicating that it is not an integral membrane protein (Figure 2C).

The secondary structure of the DoUGT71K2 protein consists of 194 residues (42.36%) in α-helices, 67 residues (14.63%) in extended strands, 23 residues (5.02%) in β-turns, and 174 residues (37.99%) in random coils, with α-helices and random coils being the predominant structural motifs (Figure 2B).

Homology modeling of the tertiary structure of DoUGT71K2 was performed using SWISS-MODEL Workspace, with A0A1S6JYF7.1.A (glycosyltransferase from *Dendrobium* hybrid cultivar) as the template. DoUGT71K2 was predicted to assemble into a homodimer, with a GMQE (Global Model Quality Estimate) score of 0.92 and a sequence identity of 89.93% (Figure 2D). These results confirm the high reliability of the homology model and further verify the high conservation of the glycosyltransferase domain between DoUGT71K2 and the template protein.

#### 3.2.2. Phylogenetic Analysis of the DoUGT71K2 Protein

**Figure 3:**
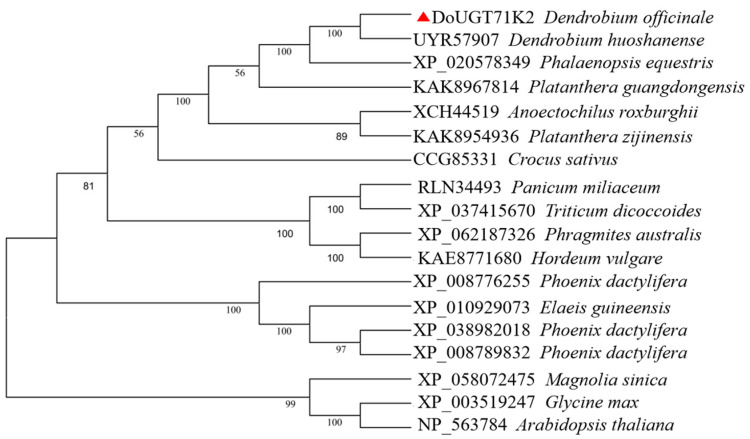
Phylogenetic relationships of DoUGT71K2 with homologous glycosyltransferases from other plant species. The red triangle indicates the DoUGT71K2 protein characterized in the present study.

Pairwise amino acid sequence identities between DoUGT71K2 and its homologous glycosyltransferases from various species varied from 44% to 65%. All these homologous proteins harbor the highly conserved PSPG-box domain, which confirms that DoUGT71K2 is a member of the glycosyltransferase superfamily. Phylogenetic analysis revealed that DoUGT71K2 clustered in the same clade as the flavonoid-3-O-glycosyltransferase UYR57907 from *D. huoshanense* (Orchidaceae), indicating a close evolutionary relationship (Figure 3).

### 3.3. Tissue-Specific and Stress-Induced Expression Analysis of DoUGT71K2 in D. officinale

#### 3.3.1. Tissue-Specific Expression of DoUGT71K2 in Response to Heat Stress

**Figure 4:**
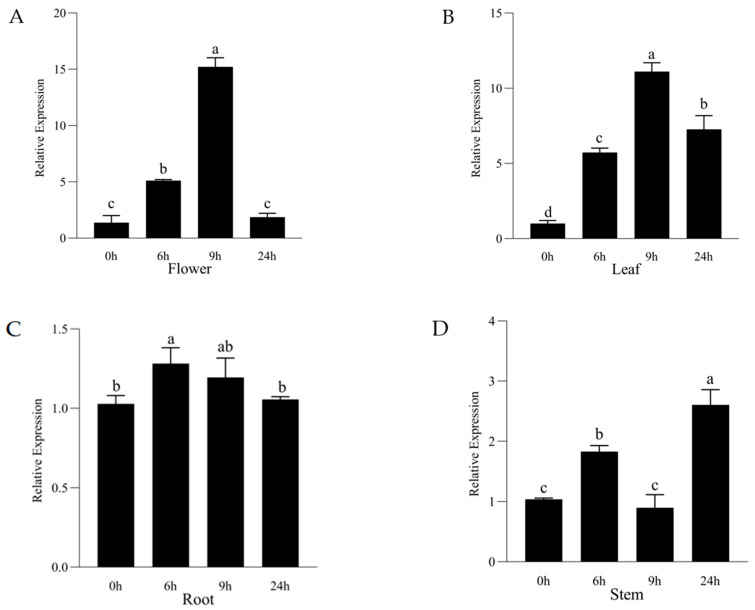
Tissue-specific expression patterns of *DoUGT71K2* in different organs of *D. officinale* under high temperature stress. (**A**) Flower; (**B**) Leaf; (**C**) Root; (**D**) Stem. The 0-h heat treatment samples served as the control for all tissues. Error bars represent standard deviation (SD) from three biological replicates (n = 3). Lowercase letters indicate statistically significant differences (The same letter indicates no significant difference between the treatment groups, and different letters indicate significant differences, *p* < 0.05).

Under heat stress (40 °C), the transcript abundance of *DoUGT71K2* was highest in flowers, followed by leaves, while transcript levels in stems and roots remained negligible. At the 9 h time point of heat treatment, the relative expression level in flowers was 15.47-fold, 13.96-fold, and 1.44-fold higher than those in stems, roots, and leaves, respectively (Figure 4). *DoUGT71K2* exhibited obvious tissue-specific expression patterns, suggesting that this gene may protect reproductive organs from heat-induced oxidative damage through glycosylation of secondary metabolites.

#### 3.3.2. Expression Profiles of DoUGT71K2 Under Diverse Stress Treatments

To better understand the transcriptional characteristics of *DoUGT71K2*, the PlantCARE online database was used to predict cis-acting elements within the 2000-bp promoter region upstream of its transcription start site. This promoter contains multiple stress-responsive cis-elements related to drought (DRE core), cold (LTR), and other environmental stresses (anaerobic response element, ARE), as well as hormone-inducible cis-elements associated with ABA (ABRE) and ethylene (ERE).

**Figure 5:**
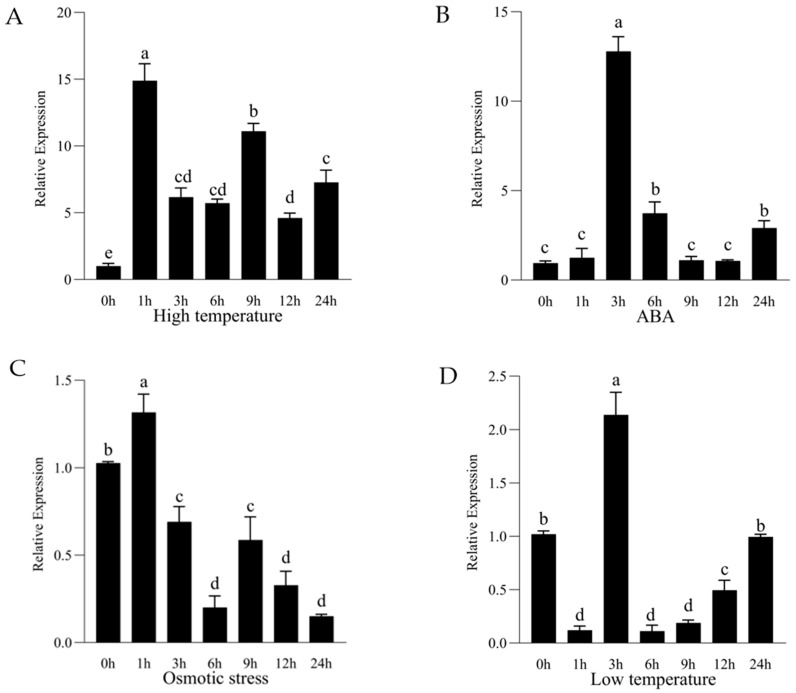
Expression profiles of *DoUGT71K2* in leaves under various stress conditions. (**A**) High temperature stress; (**B**) Exogenous ABA treatment; (**C**) Osmotic stress; (**D**) Low temperature stress. The 0-h samples were used as the controls for all four treatments. Error bars represent standard deviation (SD) from three biological replicates (n = 3). Different lowercase letters indicate statistically significant differences (*p* < 0.05).

Under heat stress at 40 °C, the transcript level of *DoUGT71K2* peaked at 1 h, whereas under cold stress (4 °C) and ABA treatment, its expression peaked at 3 h (Figure 5). The gene was significantly upregulated by heat, cold, and ABA, whereas osmotic stress had no significant regulatory effect on its transcription. These results indicate that *DoUGT71K2* exhibits stress-specific expression profiles and is presumed to be mainly involved in adaptation to temperature stress and ABA-regulated signaling pathways.

### 3.4. DoMYB102 Enhances the Transcriptional Activity of the DoUGT71K2 Promoter

The 2-kb promoter region upstream of the *DoUGT71K2* transcription start site was analyzed using the PlantCARE database to identify potential cis-regulatory elements bound by transcription factors. Several MYB-binding cis-elements, including MRE, MBS, and CCAAT motifs, were identified in the promoter sequence. RNA-seq analysis identified 12 differentially expressed MYB genes after 12 h of heat stress at 40 °C. Ten of these genes were downregulated, while only two were upregulated upon heat treatment (Supplementary Figure S1B). The two upregulated genes, *DoMYB102* (LOC110098979) and *DoMYB4* (LOC110098032), exhibited significantly increased transcript levels following heat treatment. GO enrichment analysis indicated that *DoMYB102* is involved in biological processes related to plant defense, ABA signal transduction, salt and osmotic stresses, whereas *DoMYB4* is mainly associated with cell differentiation. Based on these results, *DoMYB102* was selected for further analysis as a potential upstream regulator of *DoUGT71K2*.

**Figure 6:**
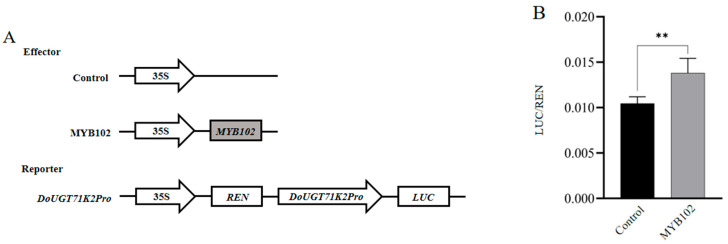
DoMYB102 activates the promoter of DoUGT71K2 as revealed by dual-luciferase reporter assay. (**A**) Schematic representation of the dual-luciferase reporter constructs. (**B**) Luminescence intensity detected in *N. benthamiana* leaves. Error bars represent standard deviation (SD) from three biological replicates (n = 3). **: *p* < 0.01.

To determine whether DoMYB102 activates the *DoUGT71K2* promoter, a dual-luciferase reporter assay was performed, with the empty pGreenII 62-SK vector as the control (Figure 6A). Co-expression of the reporter and effector constructs in *N*. *benthamiana* leaves resulted in a markedly stronger luminescence signal than that of the control (Figure 6B), indicating that DoMYB102 significantly enhances the transcriptional activity of the *DoUGT71K2* promoter.

### 3.5. DoMYB102 Binds to the DoUGT71K2 Promoter in a Y1H Assay

Two putative MYB-binding cis-regulatory elements were predicted within the *DoUGT71K2* promoter region spanning −1456 bp to −1150 bp using the PlantCare and JASPAR databases. The MBS motif (core sequence: 5′-CAACGG-3′) is located at −1456 bp to −1451 bp, whereas the stress-responsive MYB1AT motif (core sequence: 5′-AGTTAGGTTT-3′) is situated at −1331 bp to −1322 bp. To verify the physical interaction between DoMYB102 and the *DoUGT71K2* promoter, we separately amplified the 178 bp (−1331 to −1154 bp) and 307 bp (−1456 to −1150 bp) fragments harboring different combinations of these MYB-binding motifs and constructed the corresponding Y1H vectors for subsequent assays (Supplementary Figure S2).

**Figure 7:**
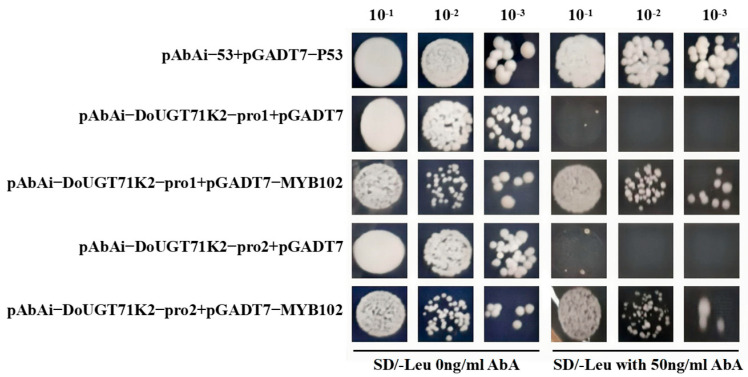
Yeast one-hybrid assay showing the interaction between the DoMYB102 transcription factor and the *DoUGT71K2* promoter fragments. Yeast cells co-transformed with DoMYB102 and the 307-bp or 178-bp promoter fragments were spotted onto SD/-Leu medium without (−) or with (+) AbA. NC: negative control (empty vector). Growth on AbA-containing medium indicates positive protein–DNA interaction.

Y1H assays were performed to further define the promoter region of *DoUGT71K2* targeted by DoMYB102. Yeast cells co-transformed with DoMYB102 and either the 307-bp or 178-bp promoter fragment grew on SD/-Leu medium supplemented with AbA, whereas the negative control did not (Figure 7). These results indicate that DoMYB102 interacts with both promoter fragments of *DoUGT71K2*. Given that the 178-bp fragment retained the ability to interact with DoMYB102, the cis-element required for binding is likely located within this region. Together, these results demonstrate a direct interaction between DoMYB102 and the *DoUGT71K2* promoter.

## 4. Discussion

*D. officinale* is well documented for its abundant accumulation of bioactive secondary metabolites, such as polysaccharides, alkaloids, and flavonoids [1,27]. Among these, flavonoids represent a crucial class of phytochemicals that not only participate in diverse physiological processes in plants but also contribute to the therapeutic effects of this species [4]. UGT-mediated glycosylation acts as a predominant modification process, which generally occurs at the final stages of flavonoid biosynthetic pathways [10]. Glycosylated flavonoids possess higher stability and solubility, which promotes their translocation and accumulation in plant cells [28]. Nevertheless, few glycosyltransferases have been functionally characterized for their catalytic activity and function in *D. officinale*. In this study, *DoUGT71K2* was identified and cloned via the transcriptome RNA-seq screening of *D. officinale* leaves subjected to 40 °C heat stress for 12 h. Conserved domain prediction revealed that DoUGT71K2 contains a conserved GT1-Gtf-like UGT domain. In addition, multiple sequence alignment of homologous glycosyltransferases from diverse species identified the characteristic C-terminal PSPG motif. These structural features classify DoUGT71K2 into the plant GT1 UGT family [29,30]. Phylogenetic analysis showed that DoUGT71K2 shares the closest evolutionary relationship with a flavonoid-3-O-glycosyltransferase from *D. huoshanense*. *AtUGT71C4*, a homolog of *DoUGT71K2*, shows significant 3-O-glycosylation activity toward the flavonol quercetin [31]. Accordingly, we speculate that DoUGT71K2 may possess similar catalytic activity and participate in synthesizing flavonoid 3-O-glycosides in *D. officinale*.

Tissue-specific expression profiling showed that *DoUGT71K2* exhibited substantially higher expression in flowers than in leaves, stems, and roots following heat treatment, suggesting its potential role in flavonoid glycosylation in floral organs under heat stress. Earlier studies have demonstrated that glycosyltransferase genes are highly expressed in floral tissues, where they mediate anthocyanin glycosylation and accumulation, which improves plant stress tolerance and regulates flower pigmentation. For example, in *Rhododendron agastum*, the 3GT Ra3GT2 glycosylates anthocyanins and flavonols, and its overexpression enhances salt and drought tolerance in transgenic *Arabidopsis* [32]. Similarly, genes encoding anthocyanin 3-O-glucosyltransferase are upregulated under low-temperature stress, and their transcript levels positively correlate with the accumulation of glycosylated anthocyanins, which results in a visible color change from white to purple in chrysanthemum (*Chrysanthemum morifolium*) [33].

*DoUGT71K2* exhibited induced expression in response to heat, cold, osmotic stress, and ABA treatment, and it displayed a rapid and strong response to high temperature and ABA, suggesting its potentially critical role in the environmental stress response of *D. officinale*. Previous studies have shown that glucosyltransferases play critical roles in enhancing tolerance to abiotic stresses. Cold stress significantly induces the expression of *CsUGT78A14* in tea, which catalyzes the glucosylation of flavonol aglycones, increasing flavonol accumulation and ROS-scavenging capacity to improve cold tolerance [19]. The glycosyltransferase UFGT2 catalyzes flavonol modification, which elevates flavonol accumulation and enhances tolerance to abiotic stresses in maize [18]. Moreover, six UGT candidates involved in flavonoid glycosylation were identified and exerted vital functions in cold adaptation of chrysanthemum [33].

Plant MYB proteins belong to one of the largest transcription factor families, which are divided into four major subgroups based on the number and spatial arrangement of conserved MYB domains: 1R-MYB, 2R (R2R3-MYB), 3R (R1R2R3-MYB), and 4R [34]. NCBI CDD conserved domain prediction revealed that DoMYB102 contains two conserved SANT/Myb_DNA-binding domains, which align with the plant-specific PLN03091 signature. This evidence places DoMYB102 into the R2R3-MYB subfamily. Members of this subfamily perform versatile biological functions, including regulation of primary and secondary metabolism, phytohormone signaling and development, as well as adaptation to biotic and abiotic stresses [35]. Plant flavonoid synthesis and metabolic transformation are intrinsically modulated by elaborate regulatory systems in response to biotic and abiotic stresses, in which transcription factors play crucial regulatory roles. Among these, R2R3-MYB proteins enhance plant stress tolerance by regulating flavonoid production [36]. MYB111, an R2R3-MYB transcription factor, promotes flavonoid accumulation by transcriptionally activating core structural genes including *CHS*, *F3H*, and *FLS*, which improve salt tolerance in *Arabidopsis* [37]. MYB112 acts as an upstream regulator of anthocyanin biosynthesis in response to salt and high-light stress in *Arabidopsis* [38]. Overexpression of MYB12/PFG1 triggers flavonoid accumulation and improves resistance to oxidative damage and drought stress in *Arabidopsis* [39].

Our study demonstrated that DoMYB102 directly binds to the *DoUGT71K2* promoter and enhances its transcriptional activity, as confirmed by two independent biochemical assays. Based on these results, we propose that DoMYB102 functions as an upstream transcriptional activator of *DoUGT71K2* under high-temperature stress, which promotes flavonoid glycosylation and accumulation and enhances the heat stress adaptability of *D. officinale*. Nevertheless, several questions remain to be addressed, including the molecular mechanisms governing the expression of *DoMYB102* itself, whether the DoMYB102-*DoUGT71K2* interaction is modulated by crosstalk with other signaling pathways (e.g., ABA and JA), and transgenic verification of its function in improving stress tolerance. Our results identify the DoMYB102-*DoUGT71K2* regulatory module as a key heat-responsive signaling cascade that potentially links flavonoid metabolism with thermotolerance in *D. officinale*.

## 5. Conclusions

We successfully cloned *DoUGT71K2*, which encodes a glycosyltransferase from *D. officinale*. Bioinformatic analysis indicated that DoUGT71K2 belongs to the GT1 UGT family and likely participates in the biosynthesis of flavonoid 3-O-glycosides. Expression profiling revealed that *DoUGT71K2* is abundantly expressed in flowers and induced by multiple abiotic stresses, particularly high temperature. Furthermore, we confirmed that the transcription factor DoMYB102, identified from transcriptome screening, activates the transcription of *DoUGT71K2* by physically binding to its promoter. These results advance our understanding of the regulatory network that links flavonoid biosynthesis with environmental stress responses in *D. officinale* and provide a theoretical basis for simultaneously improving stress tolerance and medicinal quality through molecular breeding.

## Data Availability

The raw transcriptome sequencing data generated in this study have been deposited in the Genome Sequence Archive (GSA) at the National Genomics Data Center (NGDC), Beijing Institute of Genomics, Chinese Academy of Sciences/China National Center for Bioinformation (GSA accession number: CRA047755). All raw sequencing data will be released publicly upon official publication of this manuscript. Processed expression matrices are available from the corresponding author (email: jingting_lee@lzjtu.edu.cn) upon reasonable academic request.

## Supplementary Materials

The following supporting information can be downloaded at: https://www.mdpi.com/article/10.3390/biology15151330/s1, Figure S1: Hierarchical clustering analysis of stress-responsive UDP-glycosyltransferase (UGT) family genes (A) and MYB genes in *D. officinale* identified with a cutoff of *p* < 0.05. Expression changes are presented by a color gradient ranging from blue (down-regulated) to red (up-regulated). Pale shades represent genes with small expression fold changes, whereas intense colors indicate genes with large fold changes; Figure S2: MYB-binding cis-regulatory elements predicted within the DoUGT71K2 promoter region (A) and schematic of yeast one-hybrid vector construction (B); Table S1: Detailed primer sequence information in this research.

## Abbreviations

The following abbreviations are used in this manuscript:

3GTs	Flavonoid-3-O-glycosyltransferases
4CL	4-coumarate: CoA ligase
AbA	Aureobasidin A
ABA	Abscisic acid
AS	acetosyringone
cDNA	Complementary DNA
CDS	Coding sequence
CHI	Chalcone isomerase
CHS	Chalcone synthase
DoUGT71K2	*Dendrobium officinale* UDP-glycosyltransferase 71K2
*E. coli*	*Escherichia coli*
GMQE	Global model quality estimate
JA	Jasmonic acid
LCI	Luciferase complementary imaging
LiAc/PEG	lithium acetate/polyethylene glycol
LUC	Luciferase
MIC	minimal inhibitory concentration
ORF	Open reading frame
PAL	Phenylalanine ammonia-lyase
PCR	Polymerase chain reaction
PI	Isoelectric point
PSPG	Plant secondary product glycosyltransferase
qRT-PCR	Quantitative reverse transcription PCR
Rif	rifampicin
SD	Standard deviation
UDP	Uridine diphosphate
UGTs	UDP-glycosyltransferases
UTR	Untranslated region
