# Supplementary material for: The DoMYB102–DoUGT71K2 Regulatory Module Mediates Heat-Induced Flavonoid Glycosylation in Dendrobium officinale
Source: Biology (Basel). 2026 Aug 6;15(15):1330. doi: 10.3390/biology15151330 (PMC13464734; doi:10.3390/biology15151330)
Supplement: Supplementary file 1 [file biology-15-01330-s001.zip › biology-4388720-supplementary.pdf]

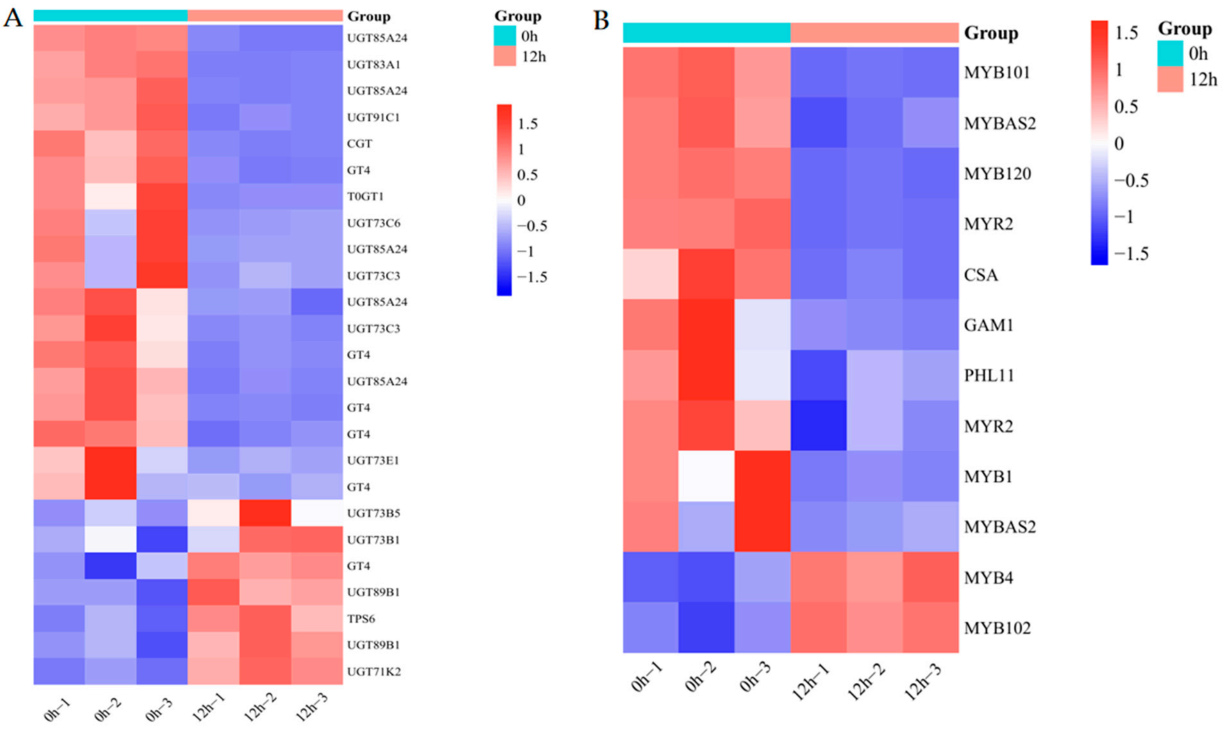

Supplementary Figure S1. Hierarchical clustering analysis of stress-responsive UDP-glycosyltransferase (UGT) family genes (A) and MYB genes *in D. officinale* identified with a cutoff of $P < 0.05$. Expression changes are presented by a color gradient ranging from blue (down-regulated) to red (up-regulated). Pale shades represent genes with small expression fold changes, whereas intense colors indicate genes with large fold changes.

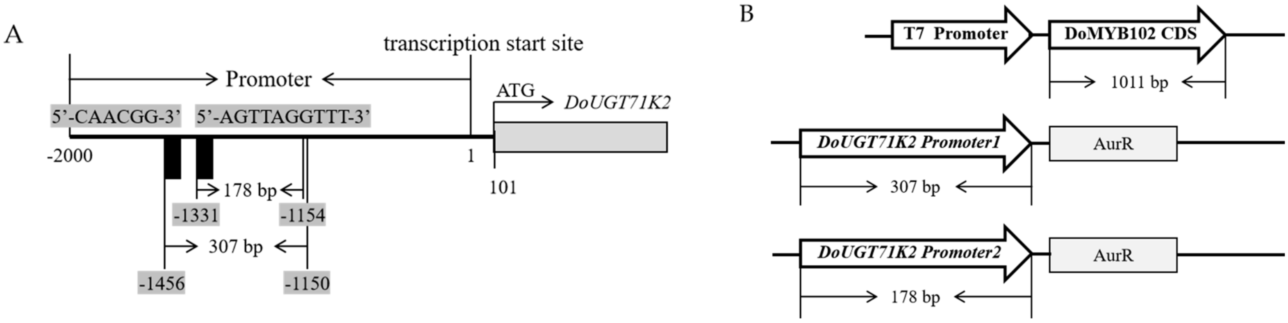

Supplementary Figure S2. MYB-binding cis-regulatory elements predicted within the *DoUGT71K2* promoter region (A) and schematic of yeast one-hybrid vector construction (B).

Supplementary Table S1. Detailed primer sequence information in this research.

Primer name	Target	product size	forward primer	reverse primer
DoUGT71K2	CDS amplification	1512	CGTCCCAAAACACGCCAAGG	TTTCCAAAGTAGCTATGCGAGAGT
MYB102	CDS amplification	1011	ATGGGAAGAGCTCCTTGCTG	TTACATGAAATCACTTACAT
Pro2K	Promoter amplification	2990	ACCAAAAGCTCATCACATAGGA	GGAGGAAGGCCAGGTTAATCTT
Actin	Real-time quantification of internal reference	150	CCCTACCTCCTACCTCTGCG	GCAAACCCAGCCTTCACCAT
5rUGT	Real-time quantitative analysis	180	CCTGTCCGTCACCATCCTTC	TAGAGTTGGAAGCAGCGAGC
Pro2K-pLUC	pGreen II 0800 Luc vector construction	2056	TCGACGGTATCGATAAGCTTAGGAGGAGAAGAAAGTAAAG	GCTCTAGAACTAGTGGATCCATATATTTTTTTCATGCAAA
MYB102-P62sk	Construction of pGreen II 62 SK vector construction	1051	GCTCTAGAACTAGTGGATCCATGGGAAGAGCTCCTTGCTG	AAGCTTATCGATACCGTCGATTACATGAAATCACTTACAT
pAbAi-DoUGT71K2pro1	pAbAi bait vector construction (for Y1H assay)	345	TGATGAATTGAAAAGCTTAGTTAGGTTTGGATGCGAAT	GATCCCCGGGTACCGAGCTCAGTTGTTGACCTCGTTTTTC
pAbAi-DoUGT71K2pro2	pAbAi bait vector construction (for Y1H assay)	216	TGATGAATTGAAAAGCTTCAACGGTCGAATACAGATTT	GATCCCCGGGTACCGAGCTCGAAAAGTTGTTGACCTCGTT
MYB102-AD	pGADT7 vector construction	1041	GAGGCCAGTGAATTCATGGGAAGAGCTCCTTGCTG	GAGCTCGATGGATCCTTACATGAAATCACTTACAT
